# Role of spontaneous ventilation in diaphragm protection during invasive mechanical ventilation in patients with acute respiratory distress syndrome: a prospective observational study

**DOI:** 10.62675/2965-2774.20260376

**Published:** 2026-07-02

**Authors:** Sangam Yadav, Pradeep Bhatia, Sadik Mohammed, Nikhil Kothari, Bharat Paliwal, Ankur Sharma

**Affiliations:** 1 Sanjay Gandhi Postgraduate Institute of Medical Sciences Department of Critical Care Medicine Lucknow India Department of Critical Care Medicine, Sanjay Gandhi Postgraduate Institute of Medical Sciences - Lucknow, India.; 2 All India Institute of Medical Sciences Anaesthesiology and Critical Care Jodhpur India Anaesthesiology and Critical Care, All India Institute of Medical Sciences - Jodhpur, India.

**Keywords:** Acute respiratory distress syndrome, Diaphragm, Length of stay, Respiration, artificial, Ventilators, mechanical

## Abstract

**Objective:**

To evaluate the effect of spontaneous ventilation duration during invasive mechanical ventilation on diaphragm protection, defined as maintaining a diaphragm thickness fraction between 15% and 30%.

**Methods:**

The present prospective, observational study enrolled one hundred adult patients with mild to moderate acute respiratory distress syndrome requiring invasive mechanical ventilation. After recruitment, the mode of invasive mechanical ventilation was recorded, and the diaphragm thickness fraction was measured daily until patients were extubated or died. Based on the total mechanical ventilation duration, patients were divided into three groups: Group 1 (3 - 7 days), Group 2 (8 - 15 days), and Group 3 (> 15 days). The patient's total spontaneous ventilation duration (more than 12 hours of ventilation on spontaneous mode comprised one day of spontaneous ventilation) was recorded in days during the mechanical ventilation stay. The duration of diaphragm protective ventilation was defined as the number of days during which the diaphragm thickness fraction remained between 15% and 30%. The primary outcome was to assess the correlation between spontaneous ventilation duration and diaphragm protective ventilation duration. The secondary outcome was the impact of spontaneous ventilation duration on total mechanical ventilation duration, intensive care unit length of stay, and survival.

**Results:**

There was a strong positive correlation between spontaneous ventilation duration and diaphragm protective ventilation duration as well as between spontaneous ventilation duration% and diaphragm protective ventilation duration% in the study population as well as in all three groups (r = 0.96, 0.91, 0.97 and 0.89, respectively; p value < 0.001) and (r = 0.88, 0.86, 0.96 and 0.90, respectively; p value < 0.001), respectively. The crude mortality rate was significantly lower with longer spontaneous ventilation duration (p value < 0.001), and a similar trend was observed in the age, SOFA score, and total mechanical ventilation duration-adjusted mortality.

**Conclusion:**

In patients with acute respiratory distress syndrome requiring invasive mechanical ventilation, encouragement of spontaneous ventilation provides diaphragm protection as evidenced by a strong positive correlation between spontaneous ventilation duration and diaphragm protective ventilation duration. In addition, patients with longer spontaneous ventilation duration may experience improved survival. Further research is needed to confirm the findings of the present study.

## INTRODUCTION

Ventilator-induced lung injury (VILI) is an iatrogenic injury to the lungs, and its significance has been recognized since the 18^th^ century; therefore, lung protection has been a priority in most ventilation strategies.^(1,2)^ The diaphragm, a highly active muscle with enormous activity during normal physiological settings, is prone to injury very easily; hence, the concept of diaphragm protective ventilation (DPV) was introduced in the 1980s by Knisely et al. in neonates.^(3)^ Ventilator-induced diaphragm dysfunction (VIDD) has been reported in 53% of patients on invasive mechanical ventilation (IMV) within 24 hours of intubation, and even a duration as short as 6 - 12 hours of IMV can lead to diaphragmatic injury.^(4,5)^ The mechanism of VIDD can be broadly classified into four major types: disuse atrophy, under-assistance myo-trauma, eccentric myo-trauma, and longitudinal atrophy.^(6-11)^

Since diaphragm injury is undesirable, several strategies have been suggested in the published literature regarding DPV, including but not restricted to encouraging a spontaneous mode of ventilation (SpV); assessing the diaphragmatic thickness fraction (DTF) as a surrogate marker for diaphragmatic activity, with values of 15 - 30% associated with early liberation from IMV; partial neuromuscular blockage in patients with acute respiratory distress syndrome (ARDS); avoidance of patient ventilator dyssynchrony; and neurostimulation, which can be given in synchrony with MV or independently.^(12-16)^ Goligher et al., in an online survey among researchers using the RAND Corporation and University of California, Los Angeles (RAND/UCLA) appropriateness rating method, suggested a few targets for lung and diaphragm protection, including monitoring inspiratory effort or respiratory drive, managing dyssynchrony, and considering sedation.^(17,18)^

Studies revealing the impact of SpV duration (SpVd) on DPV duration (DPVd) are lacking in the published literature. Therefore, the present study was conducted to assess the effect of SpVd on DPVd (the number of days during IMV that the DTF was 15 - 30%)^(14)^ in patients with ARDS requiring IMV. We hypothesized that SpVd would correlate positively with DPVd. The primary outcome of this study was to assess the correlation between the SpVd and DPVd. The secondary outcome was the impact of the SpVd on total MV duration, intensive care unit (ICU) length of stay (LOS), and survival.

## METHODS

The present single-center, single-arm, prospective observational study was conducted from September 2021 to December 2022 in the adult ICU under the Department of Anaesthesiology and Critical Care at a tertiary care center. After obtaining approval from the institutional ethics committee (Certificate Reference Number - AIIMS/IEC/2021/3672), the study was prospectively registered with the Clinical Trials Registry of India (CTRI; www.ctri.nic.in; registration no. CTRI/2021/09/037011; date of registration: Sep 30, 2021; first patient enrollment date: Oct 6, 2021). All procedures were conducted in accordance with the ethical standards of the local institutional committee on human experimentation and with the Helsinki Declaration of 2013.

Adult patients aged 18 years or older who were admitted to the ICU and receiving IMV (for more than 72 hours) through an endotracheal tube/tracheostomy tube for acute hypoxemic respiratory failure (defined as an arterial oxygen partial pressure/fraction of inspired oxygen ratio [PaO_2_/FiO_2_; P/F] < 300 with positive end-expiratory pressure [PEEP] > 5cmH_2_O) were enrolled in the study after obtaining written informed consent from the patient/legally authorized representatives. Patients who required IMV for > 48 hours in the last 6 months, have profound neurological deficits, sleep apnea, moderate to gross ascites, suspected phrenic nerve palsy, known psychiatric illness, and a history of thoracotomy were excluded.

**Figure 1:**
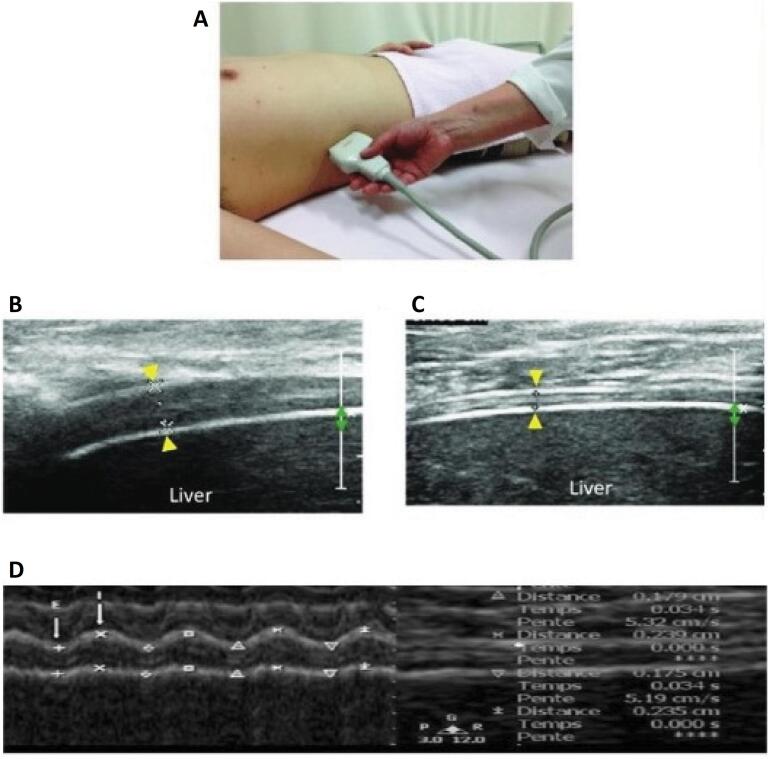
(A) Ultrasound probe position for assessment of diaphragm thickness fraction (DTF); a high-frequency linear probe (7 - 12 MHz) is placed perpendicular to the chest wall at the zone of apposition which corresponds to mid-axillary line, 8th - 11th intercostal space (B) and (C) bidimensional image of the diaphragm during inspiration and expiration respectively; (D) M-mode applied on diaphragm. E - represents the thickness of the diaphragm during expiration. I - represents the thickness of the diaphragm during inspiration.

All patients received appropriate standards of care in accordance with the ICU protocol and were ventilated with a lung-protective strategy. After enrollment, demographic characteristics, underlying disease status, reason for ICU admission, and Sequential Organ Failure Assessment (SOFA) score were recorded. The patient's ventilation mode and duration on that mode since enrollment were recorded until the patient was extubated/died. The modes of IMV for this study were primarily divided into two, namely SpV, which included pressure support (PSV) or volume support (VS); and assist control (AC) mode, which included assist control-volume control (AC-VC), assist control-pressure control (AC-PC), or synchronized intermittent mandatory ventilation (SIMV). If patients were ventilated in SpV for more than 12 hours on a given day, that day was counted as a SpV day for determining SpVd. For example, if the patient had a total MV duration of 4 days and received the AC mode of ventilation for the first 2 days and for 6 hours on day 3, after which he was on SpV for the remaining part of day 3 and day 4, the SpVd was 2 days in this patient. During the ventilation period, the DTF was recorded once daily in calm patients, defined as Richmond Agitation-Sedation Scale (RASS) scores of +1 to -1, by an experienced investigator who had received formal training in DTF measurement using ultrasound (US). The diaphragm thickness (DT) was measured (in the zone of apposition of the diaphragm and rib cage) in the midaxillary line (longitudinally) between the eighth and tenth intercostal space using a high-frequency (8-13 MHz) linear probe fitted in a US machine (Venue Go™, GE Healthcare, Chicago, United States). The diaphragm was viewed as a three-layered structure sandwiched between the two echogenic layers of the pleura and peritoneum (Figure 1A). Using the M-mode function, the thickness of the diaphragm at end inspiration (DTI) and end expiration (DTE) was measured (Figure 1B). Diaphragmatic thickness fraction was calculated by the formula [DTF = (DTI - DTE)/DTE]. Three readings were taken, and their average was recorded. If the ventilation mode changed after the measurements, we assumed DTF would remain unchanged. A DTF value of 15 - 30% was considered an indicator of DPV.^(14)^ To determine the DPVd, the total number of days on which the DTF was found to be between 15 and 30% was counted. If the DTF was > 30% or < 15%, it was not considered DPV, and the day was counted in total MV but not in DPVd. The total MV duration, SpVd, DPVd, ICU LOS, patient outcome, and cause of death were recorded for all recruited patients.

For standardization purposes, the SpVd and DPVd data were converted as percentages of total MV duration by using the formula (SpVd% = SpVd/total MV duration * 100; DPVd% - DPVd/total MV duration *100), respectively.

The impact of SpV on the diaphragm varies with the total duration of IMV: 15 days are associated with diaphragm weakness, whereas 7 days are not.^(19)^ Based on our ICU population, we decided to analyze the data based on the total MV duration as follows:

– Group 1: total MV duration 3 - 7 days
– Group 2: total MV duration 8 - 15 days
– Group 3: total MV duration 16 or more days

After the initial group division, each group was subdivided into three subgroups based on the SpVd, and the data were analyzed. This was done to account for known and unknown confounders (i.e. sepsis, critical illness neuro-myopathy etc.) which could impact our secondary outcomes.

- S50: SpVd > 50%
- S30-50: SpVd 30 - 50%
- S30: SpVd < 30%

### Statistical analysis

Sample size was calculated based on the study's primary objective, i.e., the correlation between SpVd and DPVd. As no similar literature was available, we assumed a correlation coefficient ≤ 0.35 between SpVd and DPVd as an alternative hypothesis. A sample of 83 patients would provide 5% significance and 95% power for a one-tailed hypothesis. Given a 20% attrition rate, we enrolled 100 patients.

The collected data were entered into Microsoft Excel and checked for inconsistencies. The statistical analysis was conducted using Statistical Package for Social Sciences (SPSS Inc., Chicago, IL, version 23.0 for Windows). Data normality was tested using the Kolmogorov-Smirnov one-sample test. Data were presented as median (q1, q3) for ordinal variables and for quantitative variables with non-normal distributions. Categorical variables were presented as absolute numbers or percentages. The Kruskal-Wallis test was applied to the variables to compare the three groups, since the data were nonparametric. Spearman's correlation was used to assess the relationship between two ranked variables. Logistic regression was used to identify independent predictors of survival. A p value of less than 0.05 was considered significant.

## RESULTS

**Figure 2:**
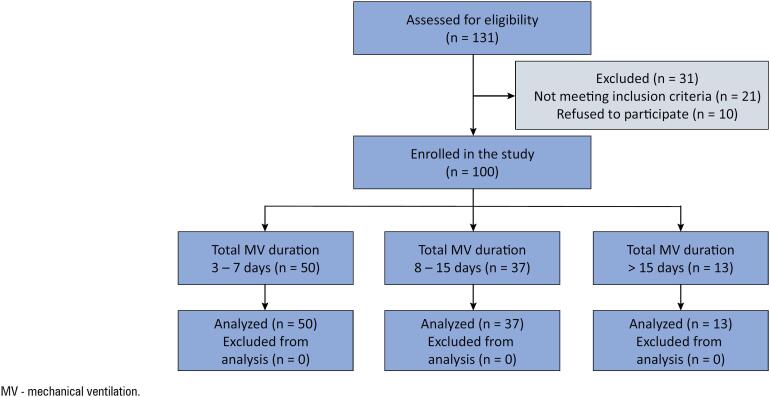
Patients’ flow during the study.

During the study period, 131 patients with acute hypoxemic respiratory failure received IMV for 72 hours or more. Of these, 31 were excluded (21 did not meet the inclusion criteria, and 10 declined to participate). The remaining one hundred patients were enrolled and followed throughout their ICU stay (Figure 2). Based on total MV duration, 50, 37, and 13 patients were assigned to Groups 1, 2, and 3, respectively. The demographic variables (age, sex, body mass index [BMI]), SOFA score, and P/F ratio at enrollment in the study population, as well as comparisons among groups, revealed no significant differences (Table 1).

**Table 1:** Demographic variables, SOFA score, and arterial oxygen partial pressure/fraction of inspired oxygen ratio in the study population and their comparison between groups

Characteristics	Study population (n = 100)	Group 1 (n = 50)	Group 2 (n = 37)	Group 3 (n = 13)	p value
Age (years)	51.5 (27, 65.8)	49.5 (25, 65)	56 (28, 66.5)	63 (43, 68.5)	0.31
Gender (M/F)	53/47	23/27	25/12	5/8	0.07
BMI (kg/m^2^)	24 (21.3, 27)	23.7 (21, 25.8)	24 (21.4, 28)	25 (22.7, 28)	0.33
SOFA score	6.5 (5, 8)	7 (6, 9)	6 (5, 8)	8 (4.5, 9)	0.18
P/F ratio at admission	234 (140, 260)	235 (129, 258)	220 (140, 268)	241 (146, 270)	0.79

M - male; F - female; BMI - body mass index; SOFA - Sequential Organ Failure Assessment; P/F - arterial oxygen partial pressure/fraction of inspired oxygen ratio. Median (q1, q3) is compared using the Kruskal-Wallis test; numbers are compared using the Chi-squared test. Data are presented as median (q1, q3) and number.

**Figure 3:**
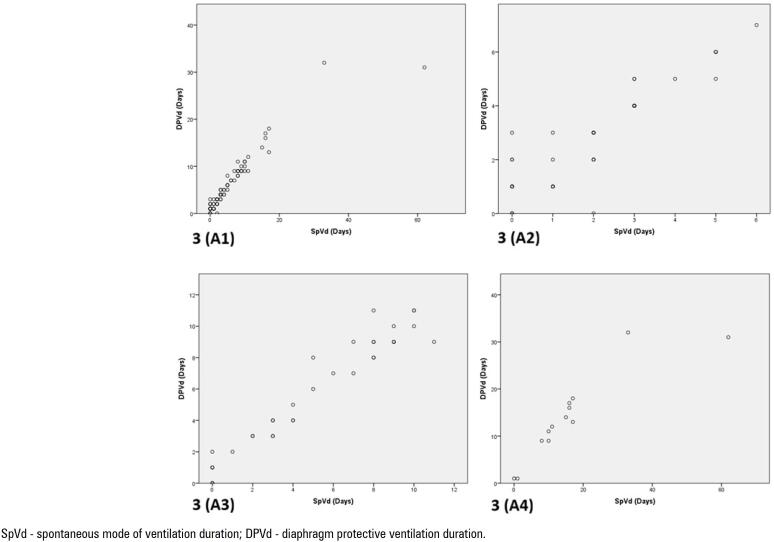
Scatter plot showing correlation between the spontaneous ventilation duration and the diaphragm protective ventilation duration in study population (3A1) and in three groups based on total mechanical ventilation duration (3A2, 3A3, and 3A4, respectively).

**Figure 4:**
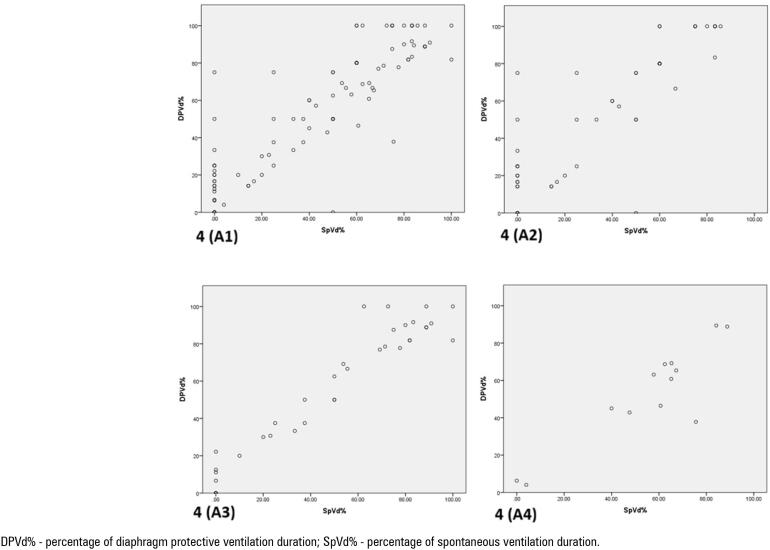
Scatter plot showing correlation between duration of spontaneous ventilation as percentage of total mechanical ventilation duration and percentage of diaphragm protective ventilation duration as percentage of total mechanical ventilation duration in study population (4A1) and in three groups based on total mechanical ventilation duration (4A2, 4A3, and 4A4, respectively).

The median (q1, q3) total MV duration of the study population was 7.5 (5, 11) days, whereas in Groups 1, 2, and 3, it was 5 (4, 6), 9 (8, 11), and 21 (18.5, 27) days, respectively. As the total MV duration (days) increased, the SpVd and DPVd (days) also increased, resulting in statistically significant differences among the groups (Table 2). However, the SpVd% and DPVd% did not differ significantly among the groups (Table 2). The effects of SpVd on DPVd and of SpVd% on DPVd% were assessed in the study population as a whole and in individual groups. A statistically significant strong positive correlation was found between SpVd and DPVd in the study population and in Groups 1, 2, and 3, with correlation coefficients (r) of 0.96, 0.91, 0.97, and 0.89, respectively (p value < 0.001) (Figure 3A1, 3A2, 3A3, and 3A4, respectively). Similarly, SpVd% and DPVd% also showed a statistically significant positive correlation in the study population and in all groups, with correlation coefficients (r) of 0.88, 0.86, 0.96, and 0.90, respectively (p value < 0.001) (Figure 4A1, 4A2, 4A3, and 4A4, respectively).

**Table 2:** Comparison of spontaneous ventilation duration, percentage of spontaneous ventilation duration, diaphragm protective ventilation duration, and percentage of diaphragm protective ventilation duration between groups

Characteristics	Study population	Group 1 (n = 50)	Group 2 (n = 37)	Group 3 (n = 13)	p value
SpVd (days)	3 (0, 7)	2 (0, 3)	4 (0.5, 8)	15 (9, 17)	< 0.001
SpVd%	50 (0, 72.4)	40 (0, 60)	50 (5, 80.9)	62.5 (43.8, 71.5)	0.07
DPVd (days)	4 (1,8)	2.5 (1, 4)	5 (2, 9)	13 (9, 17.5)	< 0.001
DPVd%	58.6 (20, 81.8)	53.6 (19.2, 80)	62.5 (21.1, 88.2)	60.8 (40.3, 70)	0.97

SpVd - spontaneous ventilation duration; SpVd% - percentage of spontaneous ventilation duration; DPVd - diaphragm protective ventilation duration; DPVd% - percentage of diaphragm protective ventilation duration. Median (q1, q3) is compared using the Kruskal-Wallis test. Data are presented as median (q1, q3).

In Groups 1 and 2, there was a weak positive nonsignificant correlation between SpVd and total MV duration. In contrast, in Group 3, the correlation was significant and strongly positive (r = 0.2, 0.25, and 0.94, respectively; p values = 0.18, 0.14, and < 0.001) (Figure 1SA - Supplementary Material). The median (q1, q3) ICU LOS among groups (1, 2 and 3) and (S50, S30-50 and S30) is included in table 1S (Supplementary Material). At S50 and S30 - 50, the ICU LOS had a statistically significant positive correlation with the SpVd (r = 0.85 and 0.74, respectively; p values ≤ 0.001 and 0.02, respectively), while at S30, the correlation was nonsignificant and weakly positive (r = 0.22; p value = 0.19) (Figure 1SB - Supplementary Material).

In the study population, survival was significantly better with increasing SpVd (73.3%, 35.3%, and 21.1%, respectively; p < 0.001). The crude mortality was 26.7%, 64.7%, and 78.9% in patients with SpVd% > 50%, 30 - 50%, and < 30%, respectively. In a multivariable logistic regression adjusting for age, baseline SOFA score, and total MV duration, SpV exposure was independently associated with reduced mortality (Table 3). Compared with patients receiving SpV < 50% of total MV duration, those receiving SpV > 50% of total MV duration had significantly lower odds [adjusted OR (95%CI); p value] of death [0.083 (0.03 - 0.28); p < 0.001]. Neither age, SOFA score, nor total MV duration was independently associated with mortality in the adjusted model (Table 3).

**Table 3:** Multivariable logistic regression analysis for adjusted outcome

Variable	Odds ratio	95%CI	p value
Age	0.98	0.96 - 1.01	0.09
SOFA score	0.90	0.75 - 1.09	0.28
S50	0.083	0.03 - 0.28	**<0.001**
Total MV duration (> 15 days)	1.36	0.35 - 5.23	0.66

95%CI - 95% confidence interval; SOFA - Sequential Organ Failure Assessment; S50 - spontaneous ventilation duration > 50% of total mechanical ventilation; MV - mechanical ventilation. For analysis purposes, the total mechanical ventilation duration and spontaneous ventilation duration were converted into two categories: total mechanical ventilation duration < or > 15 days and spontaneous ventilation duration < or > 50% of total mechanical ventilation duration.

## DISCUSSION

The present study demonstrated that in patients with acute hypoxemic respiratory failure requiring IMV, SpVd had a strong positive correlation with DPVd. The correlation was maintained across the groups with different total MV durations, as well as when the SpVd and DPVd data were presented as percentages of total MV duration. The total MV duration and ICU LOS were positively correlated with the SpVd. Age, SOFA score, and total MV duration-adjusted survival were significantly better in patients with longer SpV exposure.

Lung-protective ventilation has been widely studied and discussed in literature, making it the core of most ventilation strategies. However, the diaphragm bears the cost of these strategies, as highlighted by several studies.^(14,20)^ Karageorgos et al. and Levine et al. highlighted the significance of the SpV in preserving diaphragmatic contraction.^(21,22)^ Yoshida et al. coined the term "safe" SpV to provide diaphragm and lung protection.^(23)^ In our study, one hundred patients were observed until extubation/death, revealing the impact of SpVd on the diaphragm over a longer duration in contrast to previous trials.^(24)^ Diaphragmatic thickness fraction, being a non-invasive and moderately reliable tool, is a lucrative option.^(25)^ The strong correlation between SpVd and DPVd suggests that the SpV mode should be implemented as soon as possible to avoid further damaging the diaphragm. The positive impact of SpV on diaphragm protection was observed consistently across MV durations (Groups 1, 2, and 3). Our findings may be explained by the fact that diaphragmatic contractions are beneficial because they optimize ventilation distribution and prevent disuse atrophy. By maintaining active muscle engagement, spontaneous breathing enhances gas exchange in the dependent lung regions while protecting the diaphragm from the functional decline associated with complete mechanical support.^(21)^ Spontaneous breathing is only beneficial when carefully managed. It plays a vital role in protecting both the lungs and the diaphragm, yet it requires precise titration of effort. To avoid injury, clinicians must prevent excessively low or high inspiratory efforts as well as patient-ventilator asynchronies. Ultimately, SpV modes should be used only if proven to remain "lung-protective". This information may be useful in difficult, prolonged weaning cases using two methods. Firstly, we can diagnose VIDD if the DTF falls outside the range of 15 - 30%. Secondly, if feasible, initiating SpV can prevent further harm to the diaphragm. In essence, as achieving SpV is a necessary step before extubation, there is an inherent selection bias/survivor bias in the results, and this should be a focus for future research.

In our study, we were unable to establish a meaningful impact of SpVd on total MV duration, which mirrors the observations of Grassi et al., in which improvement in DT did not affect MV duration.^(26)^ We found a significant increase in the ICU LOS with increasing SpVd% only in Group 1. These findings could be explained by better survival with increasing SpVd%, which in turn increased ICU LOS in subgroup S50 compared to the other subgroups. Considering the broader picture across all studied patients, factors other than ventilation type (assisted or spontaneous) appear to play a much more important role in ICU LOS and the duration of total MV, particularly in patients with a long duration of MV.

We could also establish some association between improved survival and increasing SpVd. We found that survival improves with increasing SpVd across the total MV duration. Subgroup S30-50 was too small (n = 9) to reflect the true picture. There are several reasons for reduced survival, including but not limited to critical illness myopathy, secondary sepsis, severity of illness, etc. Our findings are consistent with those of Schepens et al. and Goligher et al.^(5,14)^ By analyzing the entire study population, we found that SpVd and DPVd were significantly associated with survival. The age, SOFA score, and total MV duration-adjusted survival were found to be better in patients with SpVd of more than 50% of the total MV duration.^(27)^

Our study has a few limitations. First, due to the observational design, the inherent bias (selection bias) in trial design could not be entirely excluded. Second, although initial SOFA scores were recorded, the gradual worsening of SOFA scores would have affected total MV duration and ICU LOS, which were not recorded in this study. Third, US-acquired images are only moderately reproducible; therefore, intraobserver error cannot be completely eliminated. Fourth, the operational definition of "1 day of SpV" (> 12 hours in spontaneous mode) is somewhat arbitrary and may not accurately reflect physiological exposure. Fifth, only one parameter was considered synonymous with the DPV, which would have introduced observer bias. Moreover, the assumption that DTF remains unchanged after ventilatory mode switch may not hold true in dynamic ICU settings. Sixth, we did not analyze the impact of PEEP on DPV. The inclusion of other parameters for DPV assessment, such as airway occlusion pressure and esophageal pressure monitoring, would increase the specificity of DPV detection. Also, the ventilator adjustments to allow DPV were not collected. Future studies with multicenter randomized controlled designs and larger populations, focusing on finding the balance between SpV and lung-protective goals, are required to confirm the findings of our study.

## CONCLUSION

The diaphragm should also be given due importance, as should the lungs or kidneys, and efforts to reduce ventilator-induced diaphragm injury should be implemented whenever feasible. Increasing the duration of spontaneous ventilation could be one measure for providing diaphragm-protective ventilation. Additionally, promoting spontaneous ventilation may be associated with improved survival in patients with acute hypoxemic respiratory failure requiring invasive mechanical ventilation. These observations could be used to design subsequent randomized controlled trials investigating other strategies/interventions to protect the diaphragm without compromising lung-protective ventilation, and to analyze the true impact of diaphragm-protective ventilation on survival.

## Data Availability

The datasets used and/or analyzed during the current study are available from the corresponding author on reasonable request.
