# Supplementary material for: Role of spontaneous ventilation in diaphragm protection during invasive mechanical ventilation in patients with acute respiratory distress syndrome: a prospective observational study
Source: Crit Care Sci. 2026 Jul 2;38:e20260376. doi: 10.62675/2965-2774.20260376 (PMC13399225; doi:10.62675/2965-2774.20260376)
Supplement: Supplementary Material [file 2965-2774-ccsci-38-e20260376-suppl.pdf]

Critical Care Science
https://doi.org/10.62675/2965-2774.20260376

SUPPLEMENTARY MATERIAL

Role of spontaneous ventilation in diaphragm protection during invasive mechanical ventilation in patients with acute respiratory distress syndrome: a prospective observational study

Sangam Yadav[1], Pradeep Bhatia[2], Sadik Mohammed[2], Nikhil Kothari[2], Bharat Paliwal[2], Ankur Sharma[2]

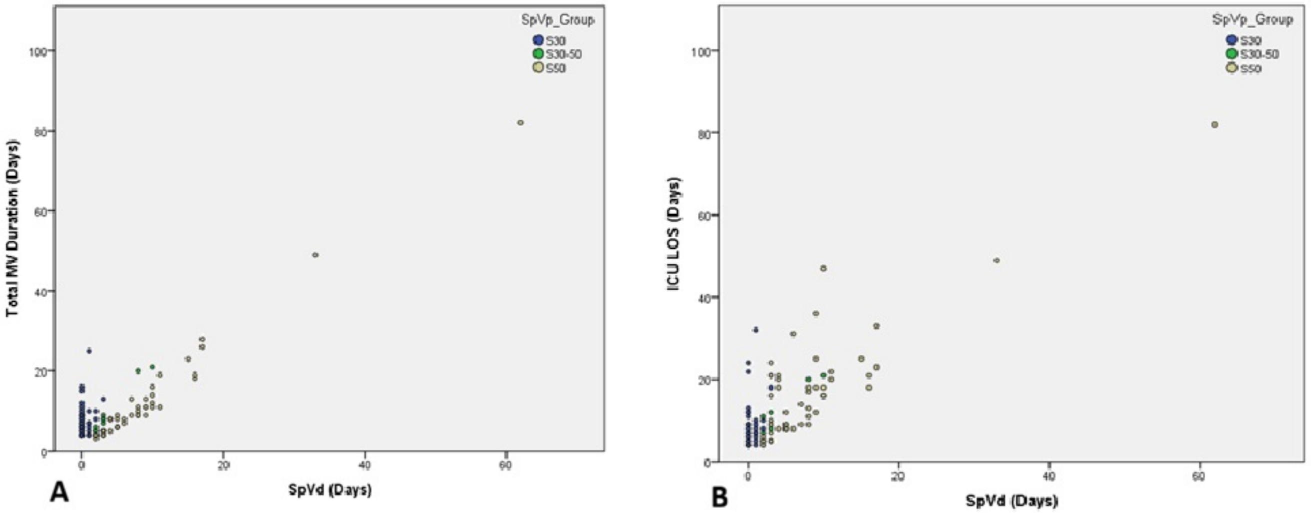

SpVd - spontaneous ventilation duration; ICU - intensive care unit; LOS - length of stay.

Figure 1S - Scatter plot showing correlation between (A) total mechanical ventilation duration (days) and spontaneous ventilation duration (days) among three groups based on total mechanical ventilation duration, (B) intensive care unit length of stay, and spontaneous ventilation duration.

Table 1S - Comparison of intensive care unit length of stay among groups

Groups	S50<br>(n = 53)	S30-50<br>(n = 9)	S30<br>(n = 38)	p value
Group 1 (n = 50)	8.5 (5 - 17.3)	5 (4.5 - 9)	6 (4 - 7)	0.01*
Group 2 (n = 37)	15 (9 - 19)	13.5 (8 - 20.3)	10 (8.5 - 12.5)	0.2
Group 3 (n = 13)	23 (19 - 41)	20.5†	27†	0.56

S50 - spontaneous ventilation duration > 50% of the total mechanical ventilation duration; S30 - 50 - spontaneous ventilation duration 30 - 50% of the total mechanical ventilation duration; S30 - spontaneous ventilation duration < 30% of the total mechanical ventilation duration. Median (q1, q3) is compared using the Kruskal-Wallis test. Data are presented as median (q1, q3). * The value is stastically significant; † there was no range for the given data set, only median.

This is an open access article under the CC BY license (https://creativecommons.org/licenses/by/4.0/).
